# The human HOXA9 protein uses paralog-specific residues of the homeodomain to interact with TALE-class cofactors

**DOI:** 10.1038/s41598-019-42096-y

**Published:** 2019-04-05

**Authors:** Amélie Dard, Yunlong Jia, Jonathan Reboulet, Françoise Bleicher, Catherine Lavau, Samir Merabet

**Affiliations:** 1 Institut de Génomique Fonctionnelle de Lyon, Université de Lyon, Université Lyon 1, CNRS, Ecole Normale Supérieure de Lyon, 46 allée d’Italie 69364, Lyon cedex, 07 France; 2 Neurosurgery, Duke University Medical Center, Research Drive, LSRC building, Room C-243, Box 91001, Durham, NC 27710 USA

## Abstract

HOX proteins interact with PBX and MEIS cofactors, which belong to the TALE-class of homeodomain (HD)-containing transcription factors. Although the formation of HOX-PBX complexes depends on a unique conserved HOX motif called hexapeptide (HX), the additional presence of MEIS induces a remodeling of the interaction, leading to a global dispensability of the HX motif for trimeric complex formation in the large majority of HOX proteins. In addition, it was shown that the anterior HOXB3 and central HOXA7 and HOXC8 proteins could use different alternative TALE interaction motifs, with or without the HX motif, depending on the DNA-binding site and cell context. Here we dissected the molecular interaction properties of the human posterior HOXA9 protein with its TALE cofactors, PBX1 and MEIS1. Analysis was performed on different DNA-binding sites *in vitro* and by doing Bimolecular Fluorescence Complementation (BiFC) in different cell lines. Notably, we observed that the HOXA9-TALE interaction relies consistently on the redundant activity of the HX motif and two paralog-specific residues of the HOXA9 HD. Together with previous work, our results show that HOX proteins interact with their generic TALE cofactors through various modalities, ranging from unique and context-independent to versatile and context-dependent TALE binding interfaces.

## Introduction

HOX genes encode for homeodomain (HD)-containing transcription factors (TFs) that are involved in the control of numerous processes during embryonic development^1^. This evolutionary conserved family of developmental regulators is classified into anterior, central and posterior paralog groups (PGs), reflecting their organization and function along the anterior-posterior (AP) axis of the embryo^2^. Mutations affecting this organization and resulting in the inappropriate expression of a HOX gene product along the AP axis lead to the famous homeotic transformations^3^. These phenotypes emphasize that each HOX protein has specific functions during embryogenesis.

HOX proteins are known to regulate distinct sets of target genes *in vivo*, which is at odds with their highly conserved HD and similar DNA-binding properties *in vitro*^4–6^. This paradox has in part been solved by the identification of the PBC-class cofactors, which belong to the TALE family of HD-containing TFs^7^. PBC representatives include Extradenticle (Exd) in *Drosophila* or PBX1-4 in vertebrates. All PBC proteins form dimeric complexes with HOX proteins from PGs 1-10^8,9^. Importantly, dimeric HOX/PBC complexes display distinct DNA-binding properties, with increased specificity and affinity when compared to the HOX monomer binding^10^. Crystal structures of several vertebrate and invertebrate HOX/PBC complexes have also shown the preponderant role of a canonical HOX peptide motif called Hexapeptide (HX) or W-containing motif (because of the presence of an invariant conserved W residue in all HX motifs) in establishing strong contacts with particular residues of the PBC HD^11–14^. More recently, the interaction between HOX and PBC was described as revealing a “latent specificity”, allowing paralog-specific residues of the HOX protein to recognize a typical shape of the DNA minor groove^14^.

Crystal structures have so far been obtained with incomplete HOX and PBC proteins, and important information could therefore be lacking. In addition, no structure has been solved in the presence of the third partner MEIS, which forms trimeric complexes with HOX and PBC proteins^15^. MEIS proteins also belong to the TALE family and are required for the nuclear translocation of PBC, a role that is evolutionary conserved in the animal kingdom^16,17^. MEIS has long been considered as a “simple” nuclear carrier of PBC but several characterized HOX target enhancers contain MEIS binding sites, highlighting that MEIS could also directly collaborate with HOX and PBC proteins on DNA^18,19^. Accordingly, MEIS forms cooperative dimeric DNA-binding complexes with PBC and with posterior mammalian HOX proteins^20,21^. MEIS was also shown to interact more generally with several HOX proteins in the absence of DNA, but the functional relevance of these interactions remains to be determined^21^.

What about HOX/PBC/MEIS complexes? Several HX-mutated HOX proteins have been described to perform PBC-dependent functions *in vivo*, which led to reconsider the HOX/PBC interaction model^22–24^. In particular, it was found that MEIS was important for revealing HX-independent interaction between PBX1 and mouse or human HOX proteins^25,26^. These results suggested that HOX proteins could contain alternative interfaces to form trimeric complexes with PBC and MEIS cofactors. It was proposed that the use of different TALE interaction motifs could be at the heart of the functional diversity and specificity of different HOX/TALE complexes *in vivo*^27,28^. This hypothesis was first supported by the identification of an alternative and specific TALE interaction motif in the *Drosophila* Hox protein Ultrabithorax (Ubx)^23,29^.

Recent work revealed alternative and specific TALE interaction motifs in the human anterior HOXB3, and central HOXA7 and HOXC8 proteins^26^. Interestingly, these motifs are used in different combinations, depending on the DNA-binding site topology and cell context, demonstrating that HOX-TALE interactions are not rigid.

**Figure 1:**
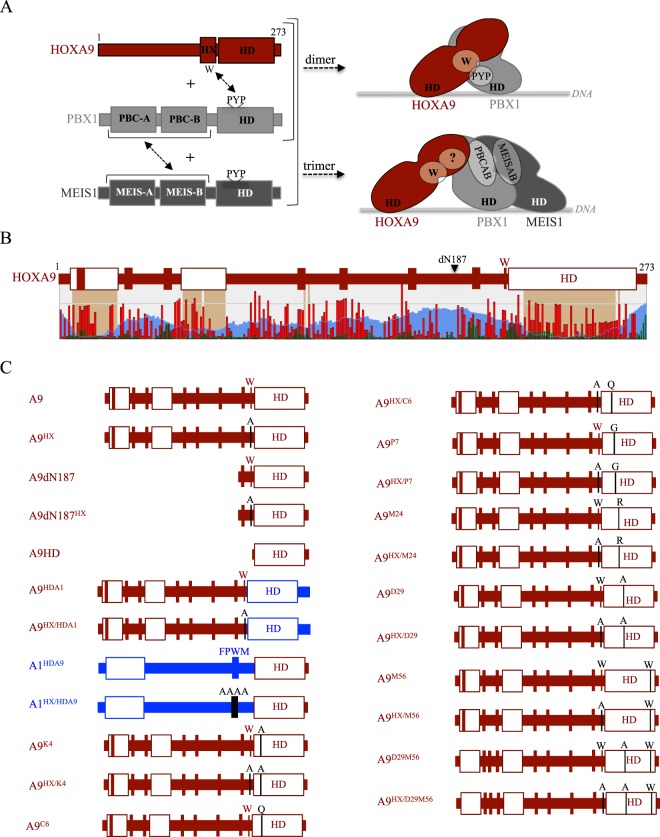
Identification of alternative TALE interaction interface(s) in the human HOXA9 protein. (**A**) Schematic representation of HOXA9, PBX1 and MEIS1, with protein domains and motifs involved in the formation of dimeric or trimeric complexes. The hexapeptide (HX) is indicated. The three PYP residues of the PBX1 homeodomain (HD) participate in the formation of an hydrophobic pocket that interacts with the conserved Trp (W) residue of the HX motif, as determined by the HOXA9/PBX1 crystal structure^13^. Domains of interaction between PBX1 (PBCA and PBCB) and MEIS1 (MEISA and MEISB) proteins are also indicated. The right panel illustrates the formation of dimeric (upper) and trimeric (lower) complexes between HOXA9 and PBX1 or HOXA9, PBX1 and MEIS1, respectively. The characterized interaction between the conserved Trp residue of the HOXA9 HX and the hydrophobic pocket formed in part by the PYP residues of PBX1 is indicated. Question mark highlights the role of additional uncharacterized binding interface(s) in HOXA9 that could be involved in the interaction with PBX1 and MEIS1 in the trimeric complex. (**B**) Schematic diagram of HOXA9 that represents predicted short linear interaction motifs (SLiMs, green bars), organized domains (brown blocks) and disordered regions (blue waves). The level of conservation of each residue among vertebrate HOXA9 sequences is also indicated (red bars). A diagram of HOXA9 summarizes the prediction of SLiMs (bars) and organized domains (white boxes). The deletion of the first 187 residues is indicated (dN187). This structure prediction was obtained with SliMPred^32^. (**C**) Schematic representation of the HOXA9 constructs analyzed with the TALE cofactors in this study. Fusions with Venus fragments are voluntarily not indicated (see also Table 1). Mutations are indicated and highlighted with a black bar. HOXA9 and HOXA1 protein fragments are in red or blue, respectively.

Here we dissected the molecular interaction properties of the human posterior HOXA9 protein with the PBX1 and MEIS1 cofactors (Fig. 1A). Compared to anterior and central HOX proteins, HOXA9 has a more divergent HD which binds DNA with higher affinity and recognizes a distinct preferential consensus nucleotide sequence^4,13^. HOXA9 also has a divergent HX motif, with a single conserved W residue, while the core Y/FPWM sequence is found in anterior and central HOX members^30,31^. Finally, HX-mutated HOXA9 was shown to interact with PBX1 in the presence of MEIS1^26^, suggesting that HOXA9 could also contain alternative TALE interaction interfaces.

We found that the formation of HOXA9-PBX1-MEIS1 trimeric complexes relied on the redundant activity of the divergent HX motif and two paralog-specific residues of the HOXA9 HD, which was obtained in the context of different DNA-binding sites and cell types. Thus, HOXA9-TALE interactions are relatively insensitive to the DNA-binding site topology and protein environment. Together with previous work, these results show that HOX proteins interact with their generic TALE cofactors through various modalities, ranging from unique and context-independent to versatile and context-dependent TALE binding interfaces.

## Results

### The homeodomain (HD) of HOXA9 is necessary and sufficient for complex formation with PBX1 and MEIS1

**Figure 2:**
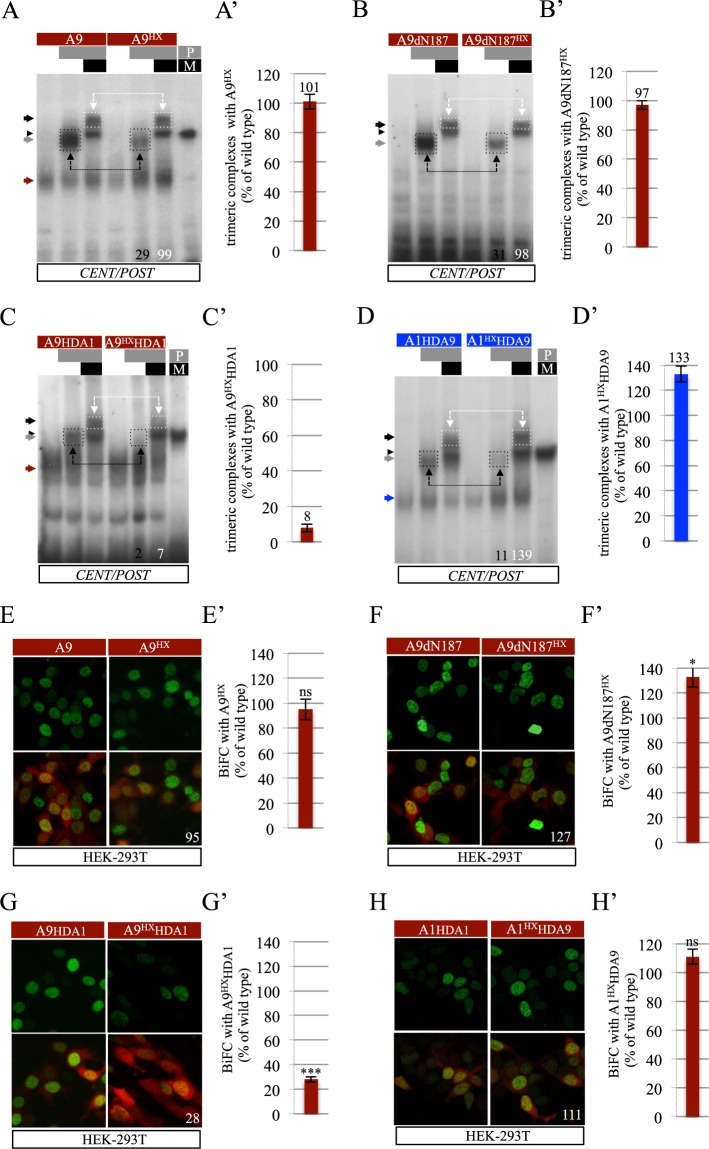
The HOXA9 homeodomain (HD) is necessary and sufficient for trimeric complex formation with PBX1 and MEIS1. **(A**–**D**) Band shift experiments of wild type or HX-mutated forms of HOXA9 with PBX1 or PBX1 and MEIS1 on the *CENT/POST* nucleotide probe, as indicated. Dimeric and trimeric complexes are highlighted (black- and white-dotted boxes, respectively). Values at the bottom indicate the quantification of dimeric and trimeric complexes as a percentage of complexes formed with the corresponding wild type HOX construct on the illustrative gel. Red/blue, black and grey arrows indicate the binding of the HOX monomere, HOX/PBX1 and HOX/PBX1/MEIS1 complexes, respectively. Black arrowhead indicates binding of PBX1(P)/MEIS1(M) complexes. (A’-D’) Quantification of trimeric complexes with the HX mutated forms of HOXA9 construct from three independent experiments (see Materials and Methods). Numbers above each bar correspond to the average value. (**E**–**H)** Illustrative confocal captures of BiFC (green) of wild type or HX-mutated forms of HOXA9 with PBX1 in HEK cells, as indicated. The quantification of HX-mutated forms is shown as a percentage of BiFC obtained with the corresponding wild type forms. The red fluorescent reporter is used to normalize results to transfection efficiency (see Materials and Methods). Note that BiFC occurs in the presence of endogenous MEIS1^26^. (E’-H’) Quantification of BiFC between the different HX mutated forms of HOXA9 and PBX1 from three independent experiments. Significance is shown relative to BiFC with the corresponding wild type form and was evaluated using t test (***p < 0,001; ns, nonsignificant). See also Supplementary Figs S1 and S2.

Previous work showed that HX-mutated HOXA9 can form a complex with PBX1 and MEIS1 on a consensus DNA-binding site called *CENT/POST* (^26^ and Supplementary Fig. S1). HX-independent interaction with PBX1 was also observed by Bimolecular Fluorescence complementation (BiFC) with PBX1 in HEK cells, which express endogenous MEIS1^26^. Given the global structure of HOXA9 (Fig. 1B), we generated a long N-terminal deletion to remove most of the predicted short peptide motifs (also called SLiMs for Short linear interaction motifs^32^), organized domains and disordered regions (Fig. 1C). The resulting 86 residues long fragment was tested either intact (construct A9dN187) or with the HX mutation (construct A9dN187^HX^). Electromobility shift assays (EMSAs) on the consensus *CENT/POST* nucleotide probe confirmed that the formation of dimeric HOXA9/PBX1 complexes but not that of trimeric HOXA9/PBX1/MEIS1 complexes was dependent on the HX motif (Fig. 2A-A’). Thus, HOXA9 can use alternative TALE interaction interface(s) in the presence of MEIS1. The A9dN187 protein fragment could also form a dimeric or trimeric complex with PBX1 or PBX1 and MEIS1, respectively (Fig. 2B-B’). Importantly, the dimeric but not the trimeric complex was strongly affected by the HX mutation, highlighting that other residues lying within the A9dN187 fragment could replace the HX motif in the presence of MEIS1 (Fig. 2B-B’).

Given that the A9dN187 fragment did not contain any obvious molecular signature with the exception of the HD, we generated another construct only containing the HD (construct A9HD in Fig. 1C). Results showed that the HOXA9 HD could strongly interact with PBX1 or PBX1 and MEIS1 (Supplementary Fig. S2).

The critical role of the HOXA9 HD for complex formation with TALE cofactors was further confirmed by generating a chimeric protein consisting of full length HOXA9 containing the HOXA1 HD, with or without the HX mutation (constructs A9HDA1 and A9HDA1^HX^ in Fig. 1C). In this context, monomer binding, dimeric and trimeric complexes could be observed on the *CENT/POST* nucleotide probe, but the HX mutation led to a loss of both dimeric and trimeric complexes (Fig. 2C-C’). This result shows that the HOXA1 HD is not able to rescue the loss of the HX motif, as observed in HOXA1^26^.

We also tested the inverse chimeric protein consisting of full-length wild type or HX-mutated HOXA1, with its HD swapped with that of HOXA9 (constructs A1HDA9 and A1 ^HX^HDA9 in Fig. 1C). Results showed that the HX-mutated chimeric protein was still able to form a trimeric complex on the *CENT/POST* probe (Fig. 2D-D’), demonstrating that the HOXA9 HD was sufficient to rescue the effect of the HX mutation in HOXA1^26^.

Interaction properties of the abovementioned constructs with PBX1 were also analyzed in live HEK cells by conducting BiFC, as previously described (^26^ and Methods). We observed that the HX mutation had no effect in the context of full length or truncated HOXA9 (Fig. 2E-E’,F-F’). In addition, BiFC with wild type and HX-mutated chimeric HOXA1-HOXA9 proteins confirmed that the HOXA9 HD was necessary and sufficient (in the context of HOXA1) for the interaction with PBX1 in HEK cells (Figs 2G-G’,H-H’ and S1). BiFC also revealed considerable reduction in interaction between HOXA9 and a mutated form of PBX1 that cannot bind DNA (Supplementary Fig. S2), showing that the formation of HOXA9/TALE complexes is DNA-binding dependent. In contrast, BiFC was not affected when HOXA9 was tested with a PBX1 mutant in which the amino acids known to interact with the HX W residue, based on previous crystal structures, were altered (PYP residues of the HD^33^, Supplementary Fig. S2). This suggests that other residues of PBX1 could be involved in the interaction with HOXA9. Finally, we also performed BiFC between wild type or HX-mutated HOXA9 and PBX1 in the presence of a siRNA targeting endogenous *MEIS* in HEK cells (as described in^26^). In this context, the HX mutation led to a significant reduction (50%) of the fluorescent signals, confirming the important role of MEIS in promoting alternative molecular interaction properties between HOXA9 and PBX1 (Supplementary Fig. S2).

Altogether, EMSAs and BiFC demonstrate that the HOXA9 HD constitutes an alternative TALE interaction interface for trimeric complex formation with PBX1 and MEIS1 cofactors.

### Paralog-specific residues of the HOXA9 HD act redundantly with the HX motif for complex formation with PBX1 and MEIS1

**Figure 3:**
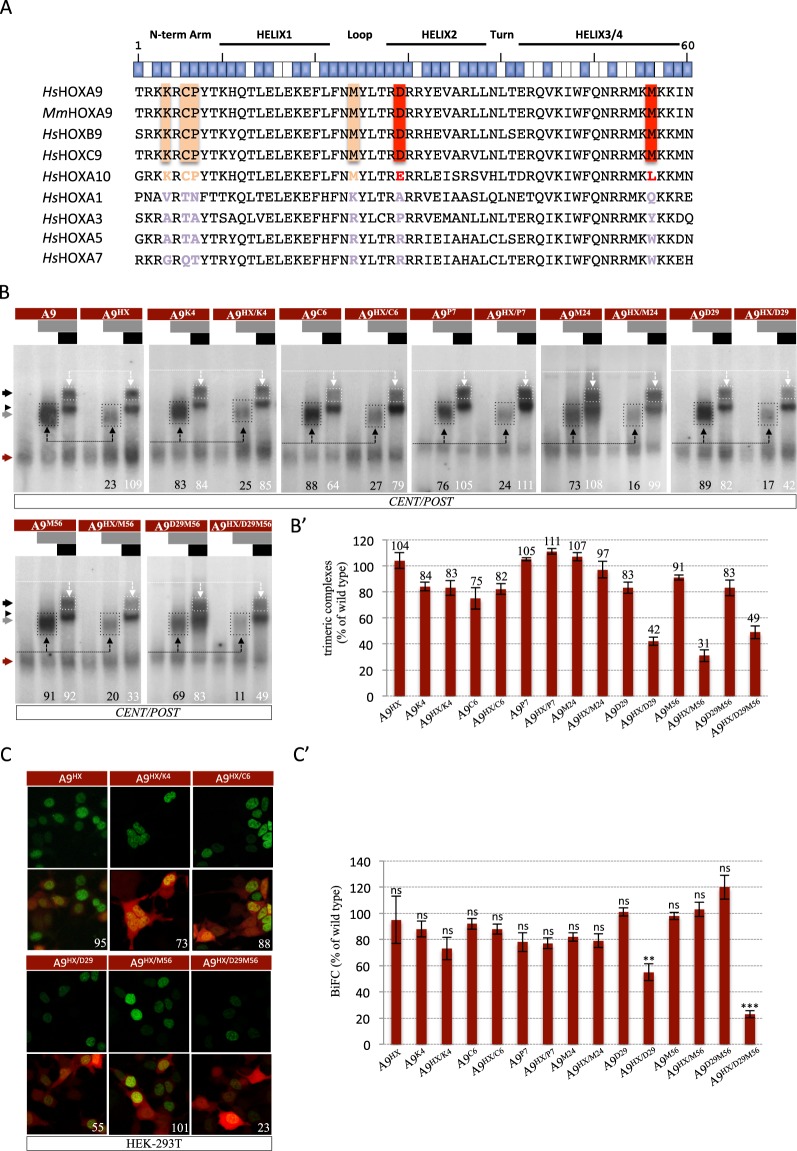
Paralog-specific residues of the HOXA9 HD are important for the interaction with TALE cofactors. **(A**) Sequence alignment of the HD of HOXA9 and other human (*Homo sapiens*, *Hs*) or mouse (*Mus musculus*, *Mm*) HOX proteins. The global structure and orientation of the aliphatic chain of each residue is indicated above the sequences. Blue and white boxes symbolize residues that are accessible or not for protein-protein interactions, respectively (based on^30^). Residues that are conserved in paralog groups 9 and 10 or only in paralog group 9 are highlighted in light orange or red, respectively. (**B**) Band shift experiments between HOXA9 constructs and PBX1, or PBX1 and MEIS1 on the *CENT/POST* nucleotide probe, as indicated. Color code and quantifications of HOXA9/TALE protein complexes are as in Fig. 2. (B’) Quantification of trimeric complexes with the different mutated forms of HOXA9 from three independent experiments. (**C**) Illustrative confocal pictures of BiFC between different HOXA9 constructs and PBX1 in HEK cells, as indicated. Color code is as in Fig. 2. (C’) Quantification of BiFC between the different mutated forms of HOXA9 and PBX1 from three independent experiments. Significance is shown relative to BiFC with wild type HOXA9 and was evaluated using t test (***p < 0,001; **p < 0,01; ns, nonsignificant).

In order to identify residues of the HOXA9 HD that could be involved in the interaction with the TALE cofactors, we performed a sequence alignment of the mouse HOXA9 HD and the HD of additional human HOX proteins from PG9 and other PGs (Fig. 3A). This analysis revealed two residues that are specifically conserved in PG9, and four residues conserved in HOX proteins of PG9 and PG10. These residues are all in positions compatible with protein-protein interactions (blue boxes above the sequences in the Fig. 3A). The two residues that are specifically conserved in PG9 proteins are located in the helix 2 and in the recognition helix (residues D29 and M56, respectively: Fig. 3A). Three out of the four residues conserved between HOXA9 and HOXA10 are located in the N-terminal arm (residues K4, C6 and P7: Fig. 3A), which is a region of the HD that is known to be important for HOX functional specificity^14^. Based on these attributes, we decided to mutate each of these conserved HOXA9 HD residues into a residue normally found in an anterior or central HOX protein (see Methods and Table 1). Mutants were analyzed in the context of wild type or HX-mutated HOXA9 (Fig. 1C) and the resulting effect on the TALE interaction potential of HOXA9 was assessed by EMSAs and BiFC, as described above.

**Table 1:** List of constructs used in this study.

Constructs	Mutations
VNHOXA9	
VNHOXA9 HX	W → *A*
VNHOXA9 DN187	
VNHOXA9 DN187HX	W → *A*
VNHOXA9 HDA1	
VNHOXA9HX HDA1	W → *A*
VNHOXA1 HDA9	
VNHOXA1HX HDA9	FDWM → F*AAA*
VNHOXA1HX HDA9D29M56	W → A **HD**: LTRDRR → LTR*A*RR RMKMKKIN → RMK*W*KKIN
VNHOXA9 K4A	**HD**: TRKKRCPYT → TRK*A*RCPYT
VNHOXA9 HX K4A	W → *A* **HD**: TRKKRCPYT → TRK*A*RCPYT
VNHOXA9 C6Q	**HD**: TRKKRCPYT → TRKKR*Q*PYT
VNHOXA9 HX C6Q	W → *A* **HD**: TRKKRCPYT → TRKKR*Q*PYT
VNHOXA9 P7G	**HD**: TRKKRCPYT → TRKKRC*G*YT
VNHOXA9 HX P7G	W → *A* **HD**: TRKKRCPYT → TRKKRC*G*YT
VNHOXA9 M24R	**HD**: FNMYLTRDRR → FN*R*YLTRDRR
VNHOXA9 M24R W	W → *A* **HD**: FNMYLTRDRR → FN*R*YLTRDRR
VNHOXA9 D29A	**HD**: FNMYLTRDRR → FNMYLTR*A*RR
VNHOXA9 HX D29A	W → *A* **HD**: FNMYLTRDRR → FNMYLTR*A*RR
VNHOXA9 M56W	**HD**: RMKMKKIN → RMK*W*KKIN
VNHOXA9 HX M56W	W → *A* **HD**: RMKMKKIN → RMK*W*KKIN
VNHOXA9 D29AM56W	**HD**: LTRDRR → LTR*A*RR RMKMKKIN → RMK*W*KKIN
VNHOXA9 HX D29AM56W	W → *A* **HD**: LTRDRR → LTR*A*RR RMKMKKIN → RMK*W*KKIN
PBX1	
CCPBX1	
PBX1 54	HD: Q -> *A*
CCPBX1 54	**HD**: Q → *A*
PBX1 PYP	HD: PYP → *AAA*
CCPBX1 PYP	**HD**: PYP → *AAA*
MEIS1a	

Residues resulting from the mutation are highlighted in italic. HD denotes residues of the homeodomain.

EMSAs on the *CENT/POST* consensus probe showed that none of the mutations in the N-terminal arm, alone or combined with the HX mutation, affected trimeric complex formation (Fig. 3B-B’). Along the same line, the M24 mutation, with or without the HX mutation, had negligible effects on complex formation with TALE cofactors, with or without the HX mutation (Fig. 3B-B’). By contrast, the D29 and M56 mutations led to a significant loss of trimeric complex formation (respectively 60% and 70% loss: Fig. 3B-B’), but only when combined with the HX mutation. This observation shows that the D29 and M56 residue act redundantly with the HX motif. Combining the HX, D29 and M56 mutations simultaneously did not further decrease trimeric complex formation (Fig. 3B-B’), suggesting that the D29 and M56 residues form two independent TALE-binding interfaces with the HX motif. The role of the D29 and M56 residues was also confirmed in the context of the HX-mutated HOXA1 chimeric protein, since their mutation strongly affected the rescue activity of the HOXA9 HD (Supplementary Fig. S3).

BiFC in live HEK cells confirmed that the three residues of the N-terminal arm and the M24 residue are not required for HOXA9-TALE interaction (Fig. 3C-C’). Also, surprisingly, the M56 mutation did not affect BiFC either (with or without the HX mutation: Fig. 3C-C’), while the D29 mutation was not neutral when combined with the HX mutation (with a global loss of 50%: Fig. 3C-C’). Combining the HX, D29 and M56 mutations led to an additive effect with a global loss of 80% of BiFC when compared to wild type HOXA9 (Fig. 3C-C’). Of note, these mutated constructs were all expressed at similar levels in nuclei of HEK cells (Supplementary Fig. S1).

Together EMSAs and BiFC show that the D29 and M56 residues of the HOXA9 HD constitute two important alternative TALE-binding interfaces that act redundantly with the HX motif.

### Role of alternative TALE-binding residues of the HOXA9 HD in different contexts

Alternative TALE interaction interfaces identified in anterior and central HOX proteins were described as being used in a highly context-specific manner with the HX motif^26^. We thus asked whether this could also apply to HOXA9. To this end, we analyzed two additional nucleotide probes for EMSAs and the HeLa and MCF7 cells for BiFC assays. The two nucleotide probes, called *CENT/POST-MEISinv* and *ANT/CENT*, diverge from the *CENT/POST* probe by containing an inversed MEIS binding site or a consensus HOX/PBX binding site for anterior and central HOX proteins, respectively (Supplementary Fig. S1 and^26^). HeLa and MCF7 cells are derived from cervical and breast cancers, respectively, as opposed to kidney derived HEK cells used above for the BiFC assays.

**Figure 4:**
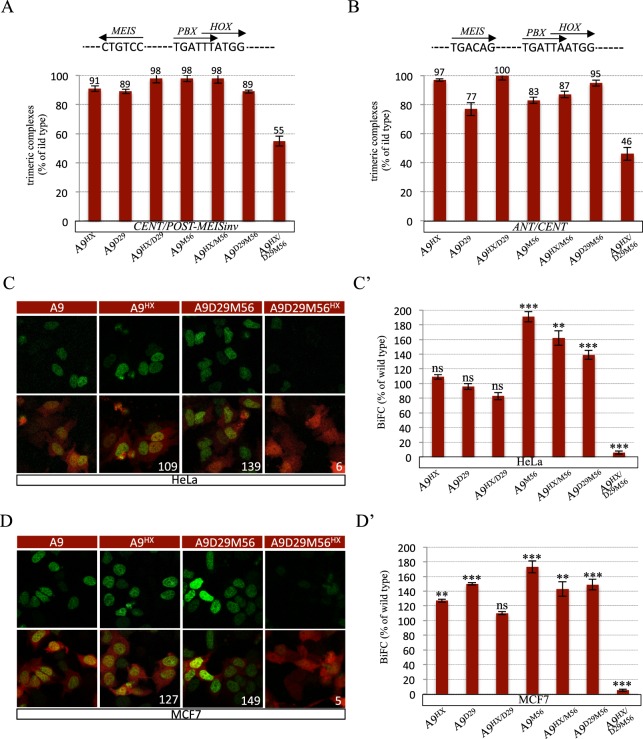
Implication of the paralog-specific residues of the HOXA9 HD for interaction with TALE cofactors in different contexts. **(A**,**B**) Quantification of trimeric complexes formed with the different HOXA9 mutants on the *CENT/POST-MEISinv* (**A**) and *ANT/CENT* (**B**) nucleotide probes as indicated. Quantifications were performed as in Figs 2. See also S3. The schematic representation of each nucleotide probe is shown on top of each gel. (**C**,**D**) Illustrative confocal pictures of BiFC between different HOXA9 constructs and PBX1 in HeLa (**C**) and MCF7 (**D**) cells, as indicated. Color code is as in Fig. 2. (C’,D’) Quantification of BiFC of the different HOXA9 mutants with PBX1 in HeLa (C’) and MCF7 (D’) cells, as indicated. Analyses were performed as in Fig. 2.

EMSAs showed that the D29 and M56 mutations only affected trimeric complex formation on the two probes when they were coupled with the HX mutation (Figs 4A,B and S3). These results demonstrate that the formation of HOXA9/TALE complexes on the *CENT/POST-MEISinv* and *ANT/CENT* binding sites relies on the redundant activity between the HX motif and the D29 and M56 residues.

BiFC in HeLa and MCF7 cells also revealed that the TALE interaction potential of HOXA9 was only significantly affected upon the simultaneous mutation of the HX motif and the D29 and M56 residues (Fig. 4C-C’,D-D’). Thus, the three TALE binding sites also behave redundantly in HeLa and MCF7 cells. Together with the previous observations, these results demonstrate that the HX motif and D29 and M56 residues are required for HOXA9-TALE interaction across different DNA binding sequences and cell contexts.

## Discussion

The identification of PBC and MEIS members as generic HOX cofactors in several developmental and oncogenic contexts has in part solved the HOX paradox to explain how a family of TFs displaying poor DNA-binding specificity *in vitro* could regulate distinct sets of target genes *in vivo*. PBC and MEIS members belong to the TALE-class of HD-containing TFs and interact with the large majority of HOX proteins. It was long considered that this interaction relied on the unique and canonical HX motif, which was somewhat at odds with the selective activity of each different HOX/TALE complex, raising the question of how HOX proteins could have different functions by interacting with the same set of TALE cofactors.

Several studies have shown that HOX proteins interact with TALE cofactors without the HX motif^26,28^, a property that is dependent on the presence of MEIS in most of the cases studied (some *Drosophila* Hox proteins being the exception:^25^). This observation indicates that HOX-PBC interactions are likely to involve considerable remodeling in the presence of MEIS. In addition, recent work identified alternative TALE interaction motifs in three different human HOX proteins, HOXB3, HOXA7 and HOXC8^26^. Interestingly, these motifs are evolutionary conserved to different extents and used independently, redundantly or even in opposition to the HX motif, depending on the DNA-binding site topology and the cell context^26^. Thus, HOX proteins can use versatile combinations of different TALE-binding motifs, showing a remarkable level of interaction flexibility with the same set of cofactors.

Here we dissected TALE interaction properties of the human posterior HOXA9 protein, which is characterized by the presence of a divergent HX motif when compared to HOX proteins of anterior and central PGs. Our results show that removal of the first 187 HOXA9 residues, which contain most of the predicted SLiMs, did not affect the interaction between HOXA9 and TALE cofactors. The HOXA9 HD was found to be sufficient to interact with PBX1 and MEIS1, not only in the context of HOXA9, but also in the context of HX-mutated HOXA1. Conversely, HX-mutated HOXA9 could not interact with the TALE cofactors when its HD was swapped with the HOXA1 HD. These observations indicate that the HD constitutes the major TALE interaction interface of HOXA9. We further demonstrated that two paralog-specific residues lying in the helices 2 and 3, D29 and M56, worked redundantly with the HX motif to promote HOXA9-TALE interactions. Mutating these two residues together with the HX motif strongly affected HOXA9-TALE interaction in MCF7 and HeLa cells but never led to a complete loss *in vitro* or in HEK cells. Along the same line, rescue of the HOXA1 HX mutation with the HOXA9 HD was not fully compromised by the additional mutation of the D29 and M56 residues *in vitro*. Thus, although the D29 and M56 residues constitute a critical TALE-binding interface, other unidentified residue(s) of the HOXA9 HD might also act as minor redundant and more context-specific TALE-binding interface(s).

The role of the D29 and M56 residues was only revealed in the presence of MEIS. The knock down of MEIS in HEK cells, or the analysis with only PBX1 in EMSA, clearly showed that the HOXA9-PBX1 interaction is fully dependent on the HX motif in the absence of MEIS, which recapitulates previous observations. The role of alternative TALE binding interfaces in the presence of MEIS raised questions about the nature of HOXA9-TALE contacts that are established in the trimeric complex. BiFC with the PYP-mutated form of PBX1 showed that the HX-binding hydrophobic pocket of PBX1 was not necessary in the presence of MEIS1. Although we cannot exclude the existence of indirect protein-protein interactions, this result again highlights the important molecular remodeling occurring between dimeric and trimeric complexes. In addition, HOXA9 can interact with MEIS1 on DNA *in vitro*^21^. Thus, both HOXA9-PBX1 and HOXA9-MEIS1 interactions have the potential to exist in the context of HOXA9/PBX1/MEIS1 trimeric complex. Given the spatial proximity between the three protagonists, the only way to get insights into the nature of HOXA9-TALE molecular contacts is by obtaining a crystal structure of the trimeric complex. This structure should be solved with at least full-length PBX1 and MEIS1 proteins to reveal all potential alternative HOXA9-TALE contacts, which remains a technically challenging issue.

The D29 and M56 residues were not identified as being important for interaction in previous crystal structures of HOXA9/PBX1 that were solved in the absence of MEIS^13^. Of note, the K58 residue of the HOXA9 HD was described as establishing a hydrogen bond with the S43 residue of PBX1 in the HOXA9/PBX1 crystal structure^13^. Interestingly, this contact is also observed in the AbdB/Exd complex, but only when the structure is solved on a particular (the highest affinity) DNA-binding site^34^. In addition, the absence of effect observed with the K4, C6 or P7 mutations is in agreement with the less important role of the N-terminal arm of the HOXA9 or AbdB HD to recognize specific minor groove width minima^34^ when compared to other Hox proteins such as Scr or Dfd^14,35^.

**Figure 5:**
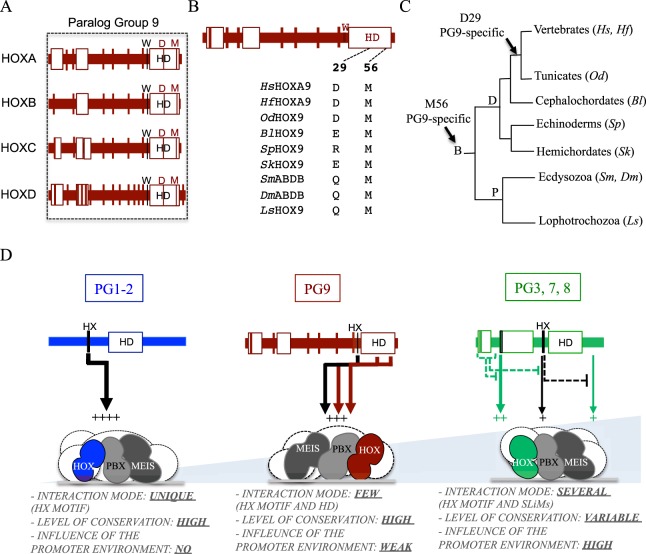
Conservation of TALE-binding residues in HOXA9 and different modes of TALE interaction among different HOX paralog groups. (**A**) The D29 and M56 residues are conserved in all human HOX members of the paralog group (PG) 9. (**B**,**C**) Conservation of the D29 and M56 residues in HOXA9 proteins from different species. The D29 residue is conserved in vertebrates and tunicates, while the M56 residue is conserved in all Bilaterian (**B**) lineages. Protein sequences were obtained from UniProt (http://www.uniprot.org). Representative Deuterostome (**D**) species are: *Homo sapiens* (*Hs*), *Heterodontus francisci* (*Hf*), *Oikopleura dioica* (*Od*), *Branchiostoma lanceolatum* (*Bl*), *Strongylocentrotus purpuratus* (*Sp*) and *Saccoglossus kowalevskii* (*Sk*). Representative Protostome (P) species are: *Strigamia maritima* (*Sm*), *Drosophila melanogaster* (*Dm*) and *Lineus sanguineus* (*Ls*). See also Supplementary Figs S4 and S5. (**D**) HOX proteins from different PGs use distinct molecular strategies to interact with TALE cofactors. A representative example is given in each case (HOXA1, HOXA9 and HOXA7). Models result from this study and from previous work^26^. The light-blue gradient illustrates the number of possible interaction modalities with TALE cofactors. HOXA1 uses a unique HX-dependent interaction mode. HOXA9 uses the HX motif and two paralog-specific residues of the HD. HOXA7 uses different alternative TALE-binding motifs in various combinations with the HX motif, depending on the DNA and/or protein environment. The importance of each TALE-binding interface within the HOX protein is symbolized by the number of signs “+” and the width of each corresponding arrow.

The D29 and M56 residues display different levels of evolutionary conservation among PG9 members: D29 is specifically conserved in vertebrates and tunicates, while M56 is conserved in all animal lineages that have been looked at (Figs 5A–C and S4). This observation suggests that the redundant role of the D29 and M56 residues as alternative TALE-binding interfaces could constitute a recent acquisition in PG9 HOX proteins during animal evolution. As a corollary, one cannot exclude the possibility that orthologous HOX9 proteins could use other alternative TALE binding interfaces in other animal lineages. Of note, whether the D29 and M56 residues are also important in human HOXB9 and HOXC9 remains to be demonstrated. Along the same line, it will be interesting to know whether HOX PG10 members could also use the same strategy to interact with the TALE cofactors. HOX10 members contain conserved residues (E29 and L56) that have similar chemical properties to D29 and M56 residues and that could therefore potentially play a similar role as the one observed in HOXA9.

The 3D modeling indicates that the D29 and M56 residues are part of two separate interaction interfaces, suggesting that their respective aliphatic chain could contact different portions in PBX and/or MEIS (Supplementary Fig. S5). Moreover, EMSA on different DNA-binding sites and BiFC in different cell contexts showed that these two residues were consistently used in redundancy with the HX motif. Thus, HOXA9-TALE interactions appear relatively insensitive to the DNA- and protein environment, which contrasts with the interaction properties of the anterior HOXB3 and central HOXA7 and HOXC8 proteins^26^. This observation highlights that different human HOX proteins use distinct molecular strategies to interact with TALE cofactors, from a unique HX-dependent interaction mode (i.e. HOX proteins from PGs 1–2), to consistently redundant (HOXA9) or versatile and context-specific (HOXB3, HOXA7 and HOXC8) TALE interaction modes (Fig. 5D). This range of molecular strategies emphasizes that HOX proteins have developed distinct levels of interaction plasticity for complex formation with their generic TALE cofactors. Understanding how these different levels of molecular plasticity could potentially be linked to functional diversity and specificity requirements of each HOX/TALE complex *in vivo* is the next challenging issue to tackle in order to crack the HOX paradox in the future.

## Materials and Methods

### Protein constructs

HOXA9 and PBX variants were generated by PCR from full-length complementary DNAs and restriction enzyme-cloned alone or in fusion with the N-terminal (VN) of Venus (for HOXA9 constructs), or the C-terminal (CC) fragment of Cerulean (for PBX1 constructs) in the pcDNA3 vector, respectively. See Table 1 for a complete list of all constructs. Complementation between VN and CC produces a Venus-like fluorescent signal, as previously described^36,37^. Primers used for constructs are available upon request. A short linker of two amino acids separates the Venus or Cerulean fragment from HOXA9 or PBX1 in all fusion constructs. The linker region corresponds to the Flag-encoding sequence in the case of the HOXA9 HD construct. The sequence of all constructs was verified before use.

### Protein Expression and Electrophoretic Mobility Shift Assays

Constructs cloned in the pcDNA3 vector were produced with the TNT-T7-coupled *in vitro* transcription/translation system (Promega). Production yields of wild type and mutated counterpart proteins were estimated by 35S-methionine labelling. EMSAs were performed as described previously^26^. Shortly, between 3 and 6 microliters of programmed lysate was used for each protein (100 ng/υl of proteins were produced on average). PBX1 and MEIS1 were co-produced together (0,5υg of each plasmid was used for the *in vitro* transcription/translation reaction). Supershift against the flag-tagged HD was performed by adding the anti-Flag antibody after 15 minutes in the binding reaction. Each band shift experiment was repeated at least three times and the quantification of protein complexes was done by using the histogram function of ImageJ software, using the complex with wild type proteins on the same gel as the reference value. Significance for each average quantification value could not be calculated because of the too small number (three to four) of samples that are considered.

### BiFC analysis in live cells

BiFC in human HEK, HeLa and MCF7 cells was performed as previously described^26^. Briefly, transfections were carried out using the JetPRIME reagent (Polyplus), with a total amount of 2 μg of DNA: 500 ng of the VN-HOX fusion vector, 500 ng of the VC-PBX1, and 1 μg of the pCMV-mCherry. Coverslips were taken 20 h after transfection, which allows having fluorescence level below saturation with each tested HOX construct. Analysis was performed with a Zeiss LSM780 confocal microscope. Pictures correspond to the Z projection of stacks, using the Zen software. Four to six different fields of cells were acquired under the same confocal parameters at the 20x objective from three independent experiments in each condition. Quantification of green (BiFC) and red (for transfection efficiency) fluorescence in all nuclei of each acquired field was realized by using the histogram function of ImageJ software. A mean ratio of green/red signals was established for each acquisition. Values obtained with wild type proteins were used as the reference for quantification.

### Immunostaining

Rabbit anti-GFP (Invitrogen A11122, 1/500) antibody was used to quantify nuclear expression of the different VN-HOXA9 fusion constructs in cell culture. Fluorescent labelling was done with an anti-rabbit (ThermoFisher A32732, 1/500) secondary antibody coupled to Alexa fluor 555.

## Supplementary information

Supplementary Information
