# Supplementary material for: The human HOXA9 protein uses paralog-specific residues of the homeodomain to interact with TALE-class cofactors
Source: Sci Rep. 2019 Apr 5;9:5664. doi: 10.1038/s41598-019-42096-y (PMC6450960; doi:10.1038/s41598-019-42096-y)
Supplement: Supplementary file 1 — Supplementary Information [file 41598_2019_42096_MOESM1_ESM.pdf]

The human HOXA9 protein uses paralog-specific residues of the homeodomain to interact with TALE-class cofactors

Amélie Dard[1,#], Yunlong Jia[1,#], Jonathan Reboulet[1,#], Françoise Bleicher[1,#], Catherine Lavau[2] and Samir Merabet[1,*].

SUPPLEMENTARY INFORMATION

Supplementary Figure S1. A. Sequence of the nucleotide probes used for the band shift experiments. The orientation of HOXA9, PBX1 and MEIS1 binding sites is indicated. The two central nucleotides of the HOX/PBX binding sites involved in DNA-binding preferences are in bold. **B-C.** Immunostaining of the HOXA9-HOXA1 chimera with an anti-GFP (green) recognizing the VN fragment at the N-terminus. **D.** Immunostaining of mutated HOXA9 constructs, as indicated. Plasmids were co-transfected with a mCherry encoding vector to assess for transfection efficiency in each condition (red). Graphs on the right show quantification of the immunostaining with the different mutated forms. Quantification is relative to the immunostaining with the corresponding wild type protein and takes into account transfection efficiency (ns: nonsignificant).

Supplementary Figure 2. A. Band shift experiment with the homeodomain (HD) of HOXA9 and the PBX1 and MEIS1 cofactors. Symbols are as in Figure 2. Asterisks denote supershifts with an antibody (γ) recognizing the Flag epitope fused to the HD of HOXA9. The two bands correspond to shifted HD/PBX1 or HD/PBX1/MEIS1 complexes. **B.** Illustrative confocal captures of BiFC between the HD of HOXA9 and PBX1 in HEK cells. The quantification is normalized to BiFC obtained with full length HOXA9 (ns:nonsignificant). **C.** Illustrative confocal captures of BiFC between HOXA9 and PBX1 mutated in the residue 54 of the HD in HEK cells. The quantification is noirmalized to BiFC obtained with wild type PBX1 (**p<0,01). **D.** Illustrative confocal captures of BiFC between HOXA9 and PBX1 mutated in the residue PYP of the HD in HEK cells. The quantification is normalized to BiFC obtained with wild type PBX1. **E-F.** Illustrative confocal captures of BiFC of wild type or HX-mutated HOXA9 with PBX1 in condition of controlled RNAi (E) or RNAi against endogenous *MEIS* in HEK cells (see Materials and Methods and [26]). The quantification is normalized to BiFC obtained with HOXA9 (***p<0,001, ns: nonsignificant).

Supplementary Figure 3. A. Band shifts experiments of chimerix HX-mutated HOXA1 proteins with PBX1 and MEIS1 on the *CENT/POST* nucleotide probe, as indicated. Note that the D29 and M56 mutations strongly affect the rescue efficiency of the HOXA9 HD in the HX-mutated HOXA1 chimeric protein. **A'.** Quantification of the trimeric complex with the HD-mutated form on the *CENT/POST* nucleotide probe. **B.** Band shifts experiments of wild type and mutated HOXA9 constructs with PBX1 or PBX1 and MEIS1 on the *CENT/POST-MEISinv* nucleotide probe, as indicated. **C.** Band shifts of wild type and mutated HOXA9 constructs with PBX1 or PBX1 and MEIS1 on the *ANT/CENT* nucleotide probe, as indicated. Color code and symbols are as in Figure 2.

Supplementary Figure 4. Alignment of HOXA9 HD sequences from different vertebrate and invertebrate species. Protein sequences were obtained from UniProt (http://www.uniprot.org). Representative Deuterostome (D) species are: *Homo sapiens* (*Hs*), *Heterodontus francisci* (*Hf*), *Oikopleura dioica* (*Od*), *Branchiostoma lanceolatum* (*Bl*), *Strongylocentrotus purpuratus* (*Sp*) and *Saccoglossus kowalevskii* (*Sk*). Representative Protostome (P) species are: *Strigamia maritima* (*Sm*), *Drosophila melanogaster* (*Dm*) and *Lineus sanguineus* (*Ls*). Sequence alignment was obtained with CLUSTALW (https://www.genome.jp/tools-bin/clustalw).

Supplementary Figure 5. Position and orientation of paralog-specific residues of the HOXA9 HD involved in the interaction with TALE cofactors. A-A'. Side chain positioning of the D29 and M56 (PG9-specific), and K4, C6 and P7 (PG9/10-specific) residues (all highlighted in orange) in two differently rotated HOXA9 HDs. The Trp (W) residue of the HX motif lying upstream of the HD is indicated in one orientation (right panel). **B-C.** Side chain positioning and chemical properties of wild type (B) or mutated (C) D29 and M56 residues (surrounded by a dotted circle). Non-charged/hydrophobic residues are in white, positively and negatively charged residues are in red or blue, respectively. The position, orientation and chemical properties of the D29 and M56 residues of the HD suggest that they could be involved in different interaction interfaces with TALE cofactors.

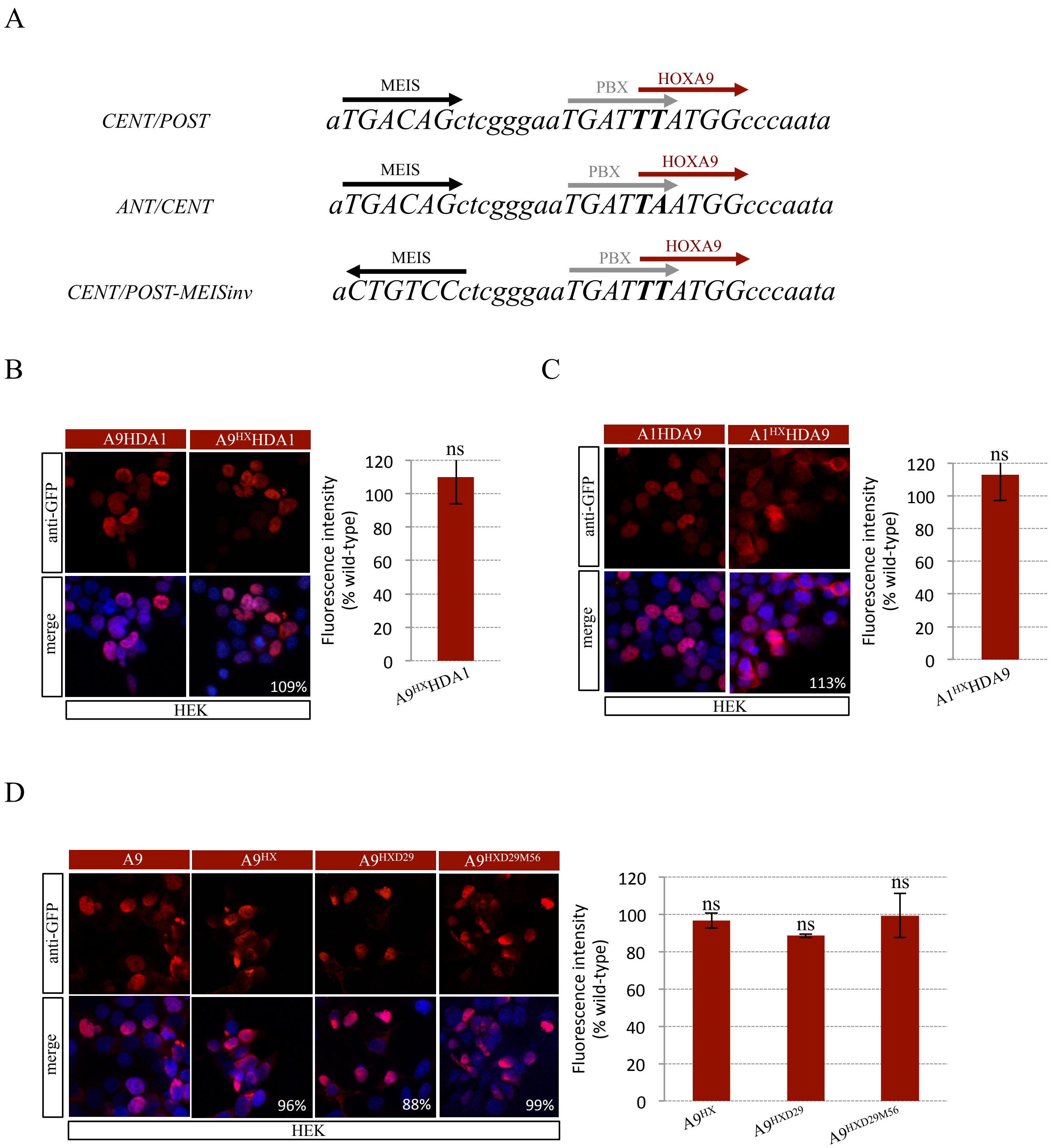

Supplementary Figure S1

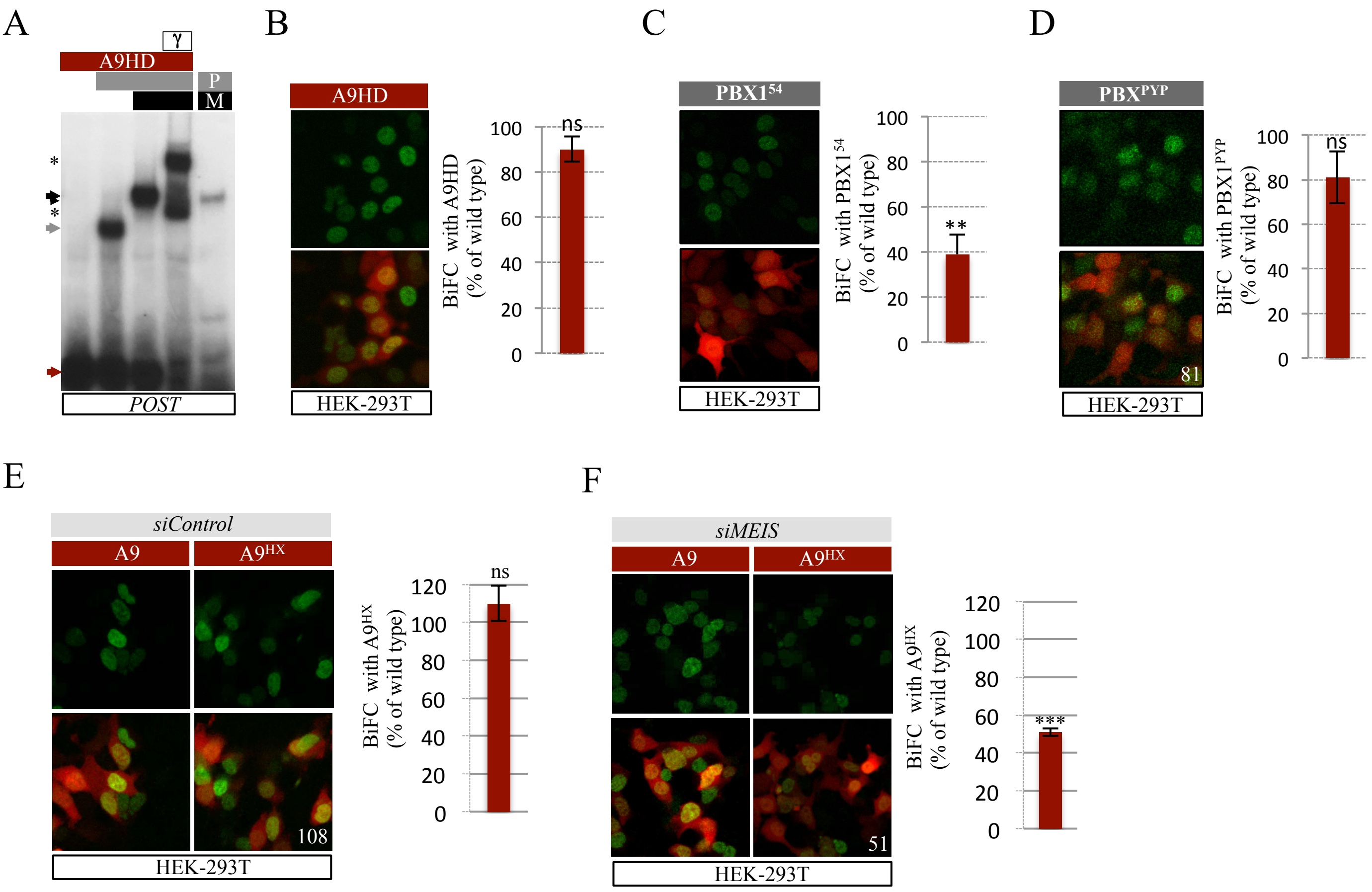

Supplementary Figure S2

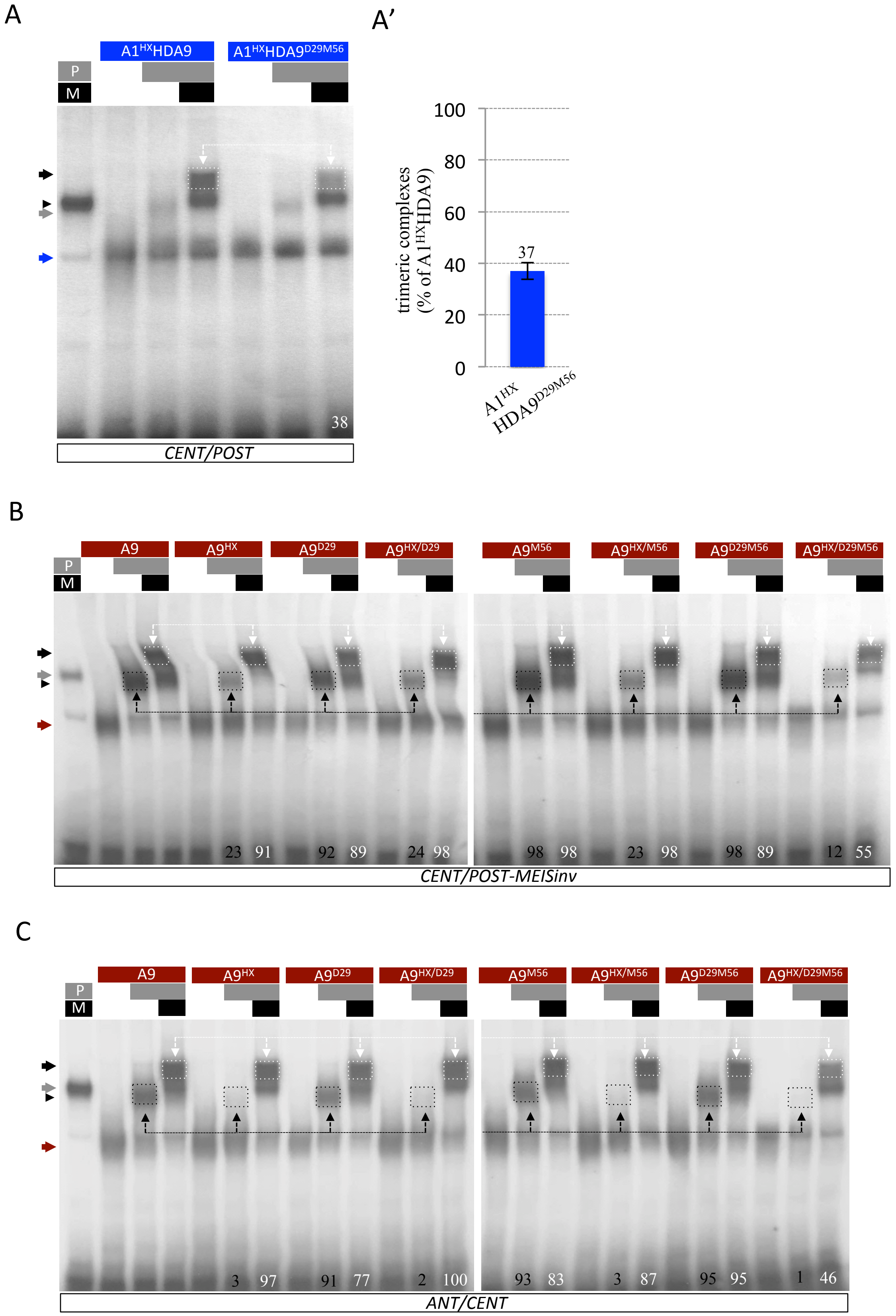

Supplementary Figure S3

```
HsHOXA9  TRKKRCPYTKHQTLELEKEFLFNMYLTRDRRYEVARLLNLTERQVKIWFQNRRMKMKKIN
HfHOXA9  TRKKRCPYTKHQTLELEKEFLFNMYLTRDRRYEVARVLNLTERQVKIWFQNRRMKMKKIN
OdHOX9   QRKKRVPYSRTQLLELEKEFRFNQYLSRDRRLELASLVNLTDRQVKIWFQNRRMKWKRER
BlHOX9   SRKKRCPYTRFQTLELEKEFLYNMYLTRERRYEISQHVNLTERQVKIWFQNRRMKMKKMS
SpHOX9   RKRCRQTYTRYQTLELEKEFHFNRYLTRRRRIELSHLLGLTERQIKIWFQNRRMKYKKES
SkHOX9   GRKKRCPYTKFQTLELEKEFLFNMYLTRERRVDIARLLNLTERQVKIWFQNRRMKLKKQN
SmAbdB   VRKKRKPYSKFQTLELEKEFLFNAYVSKQKRWELARNLNLTERQVKIWFQNRRMKNKKNS
DmAbdB   VRKKRKPYSKFQTLELEKEFLFNAYVSKQKRWELARNLQLTERQVKIWFQNRRMKNKKNS
LsHOX9   TRKKRKPYTRYQTMVLENEFLTNSYITRQKRWEISCKLHLTERQVKVWFQNRRMKRKKLN
          :: * .*:: * : **:**  * *::: :* :::  : **:**:*:******** *:
```

Supplementary Figure S4

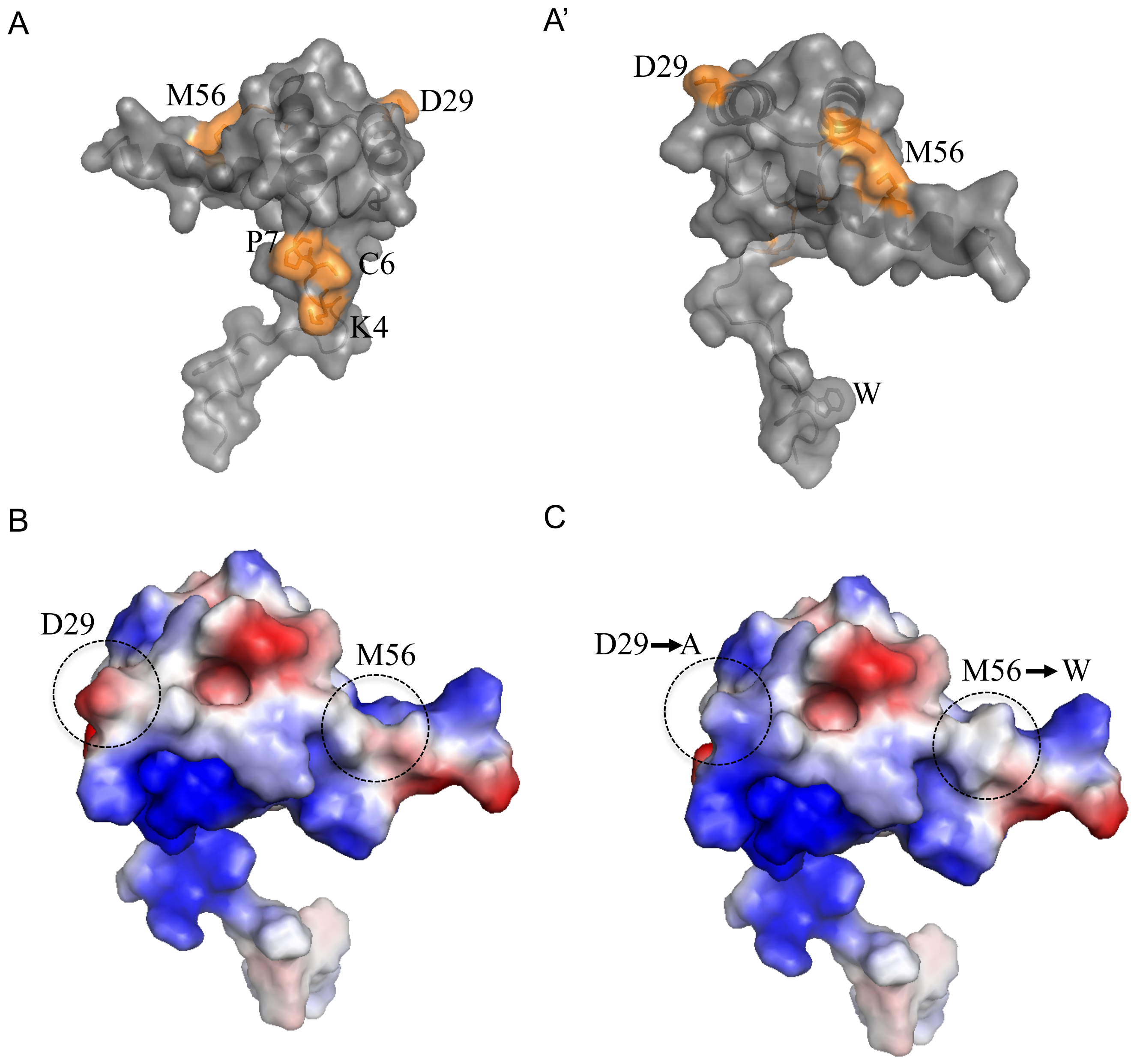

Supplementary Figure S5
